# The complete mitochondrial genome of the sand-hopper *Trinorchestia longiramus* (Amphipoda: Talitridae)

**DOI:** 10.1080/23802359.2019.1623100

**Published:** 2019-07-10

**Authors:** Ajit Kumar Patra, Min-Seop Kim, Tae Won Jung, In-Young Cho, Moongeun Yoon, Jeong-Hyeon Choi, Youngik Yang

**Affiliations:** a National Marine Biodiversity Institute of Korea, Seocheon-gun, South Korea;; b National Institute of Ecology, Yeongyang-gun, South Korea

**Keywords:** Sand-hopper, Talitridae, mitochondria, phylogeny, *Trinorchestia longiramus*

## Abstract

The complete mitochondrial genome of sand-hopper *Trinorchestia longiramus* was analyzed in this study, which is the first for the genus within the family Talitridae. The mitogenome sequence is 15,401 bp in length containing two ribosomal RNA genes, 22 transfer RNA genes, 13 protein-coding genes, and a control region as found in most amphipods. The gene order showed that *T. longiramus* has a unique control region location compared to other amphipods. Phylogenetic analysis using the maximum likelihood method positioned *T. longiramus* within the monophyletic clades of the family Talitridae.

The sand-hopper *Trinorchestia longiramus* is mostly found around South Korea (Woo et al. 2016) and also found from the northern Hokkaido to southern Tohoku in Japan (Sasago 2011). This talitrid species mostly inhabits in sandy beaches and is considered as an important trophic link between primary producers and higher consumers, helps in the energy flow within these ecosystems (Jeong et al. 2009). Mitochondrial genome (mitogenome) based phylogenetic analysis would improve our understanding of the evolutionary relationship with talitrids. However, only two talitrid mitogenomes i.e. *Platorchestia parapacifica* and *Platorchestia japonica* are available. In this study, we sequenced and analyzed the complete mitochondrial DNA sequence of *T. longiramus*. This is the first complete mitogenome for the genus *Trinorchestia* within the family Talitridae.

*Trinorchestia longiramus* were captured by hand from sandy beach (37°41′29″N, 129°2′2.7″E) of South Korea. The specimen is available with the storage number CR00246482 at the National Marine Biodiversity Institute of Korea. DNA was isolated from the specimens by conventional phenol-chloroform method (Sambrook et al. 1989). Approximately 32 Gb of reads were generated by paired-end (PE) sequencing with 251 bp read length performed in an Illumina HiSeq 2500 sequencer. Mitogenome was assembled from the PE reads using NOVOPlasty (Dierckxsens et al. 2017), where *P. parapacifica* (GenBank accession MG010371) used as the seed sequence. The mitogenome was annotated using MITOS webserver (Bernt et al. 2013); some genes were annotated manually. The mitogenome was submitted to NCBI GenBank and is available with accession number MH542431. *Trinorchestia longiramus* mitogenome is 15,401 bp in length containing 13 protein-coding genes (PCGs), two ribosomal RNAs (rRNA), 22 transfer RNAs (tRNAs), and a control region (CR), common in all amphipods.

A unique gene arrangement pattern observed in *T. longiramus* as well as in the family Talitridae, in which trnL2 is found between Nad2 and Nad3, different from amphipods (Romanova et al. 2016). Generally, the CR is located between small rRNA and Nad2 in pancrustacea mt genomes (Kilpert and Podsiadlowski 2006). However, in *T. longiramus*, CR is found between 16S and 12S rRNA genes, which is unique among all amphipods. Among PCGs, four (CytB, Cox3, Nad3, and Nad4) started with ATG, four (Cox2, Atp6, Atp8, and Nad6) started with ATA, two (Nad2 and Nad4L) started with ATT, one (Cox1) with ATC, one (Nad5) with GTG, and one (Nad1) with TTG, which have been found as start codons in crustacean mitogenomes (Lavrov et al. 2000). Ten genes ended with TAA as stop codon, two (Nad1 and Nad5) ended with TAG, and one (Cox2) ended with T(AA). In Cox2, TAA stop codon is completed by the addition of A residues to the mRNA. The overall base composition is 37.0% for A, 34.2% for T, 10.4% for G, and 18.4% for C.

A maximum likelihood phylogenetic analysis with 45 amphipod mitogenomes placed *T. longiramus* among talitrids with bootstrap value = 100% (Figure 1). *Trinorchestia longiramus* formed monophyletic clade with other *Platorchestia* species. Further study with diverse taxonomic sampling from the talitrids will help in understanding phylogenetic status of the family.

**Figure 1.**
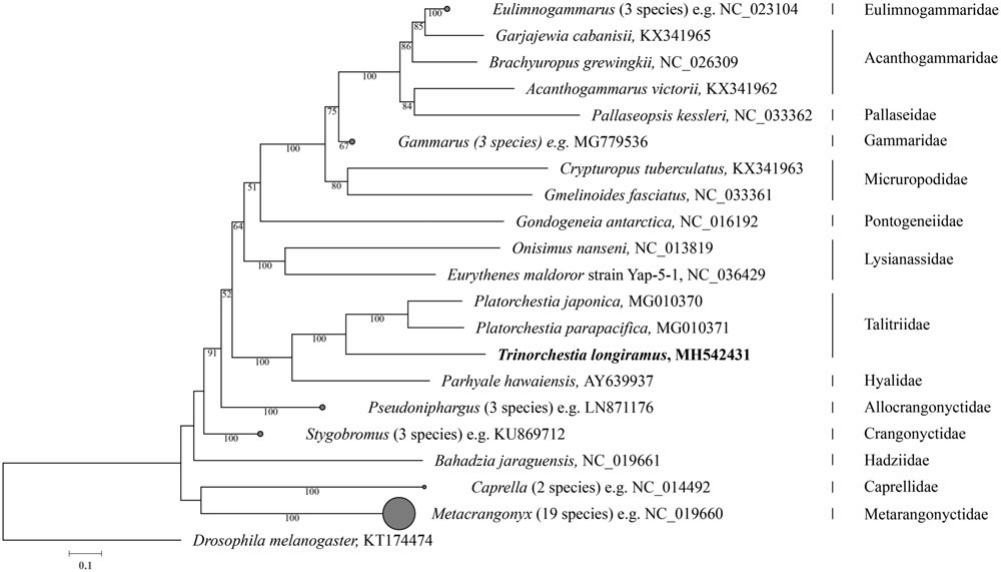
Maximum-likelihood (ML) phylogeny of 45 amphipods analyzed with the aligned and concatenated nucleotide sequences 13 protein-coding genes (11,984 bases). *Trinorchestia longiramus* is shown in bold. Sequences were aligned separately for each gene by MUSCLE v3.8.425 (Edgar 2004). ML tree was generated with RAxML v8.2.11 (Stamatakis 2014) using GTR + Γ +I substitution model supported by 1000 bootstrap replicates.
